# Cassava production as a climate change adaptation strategy in Chilonga Ward, Chiredzi District, Zimbabwe

**DOI:** 10.4102/jamba.v9i1.348

**Published:** 2017-04-25

**Authors:** Tambudzai Mupakati, Vincent I. Tanyanyiwa

**Affiliations:** 1 United Nations Development Programme, Strengthening National Capacity for Climate Change Programme, Ministry of Environment, Water and Climate, Zimbabwe; 2 Department of Geography and Environmental Studies, Zimbabwe Open University, Zimbabwe

## Abstract

This study sought to pilot a range of long-term adaptation measures in the agriculture sector because of climate change shocks. Past droughts in Zimbabwe have had devastating environmental and socio-economic impacts in rural areas where livelihoods mainly depend on agriculture. Over the past few years, many parts of Zimbabwe have been experiencing extreme events. The study sought to address the following objectives to describe smallholder farmers’ knowledge of climate change variability and change in Chilonga Ward and to explore the potential of cassava production as a climate change adaptation strategy in Chiredzi. An assessment of the impact of cassava production on rural livelihoods as a climate change adaptation strategy was also done. Focus group discussions, in-depth interviews, desk research and observation were the tools used to collect data. The results show that cassava has an extensive root system that can penetrate poor soils which may not support crops like maize. Zimbabwe has to increase cassava production as its tubers can be value added to produce a range of products that include livestock feed and porridge.

## Introduction

One of the key challenges facing humanity today is climate change. The Fourth Assessment Report of the Intergovernmental Panel on Climate Change (IPCC 2007, 2010) indicates that the energy balance within the Earth’s climate system has been altered, and this has resulted in significant changes in national, regional and world climate. IPCC (2007) has observed increases in average air and ocean temperatures, widespread melting of snow and ice, and rising global average sea level. In Zimbabwe, climate change presents risks to lives and livelihoods at the individual level and to the economy, and the livelihoods of the poor are highly vulnerable to climate change because of heavy reliance on rain-fed agriculture (Brown 2011). Zimbabwe has not been spared from the effects of climate change (Downing 1991). The country’s annual mean surface temperature has warmed by about 0.4 °C from 1900 to 2000 (Chigwada 2009). The occurrence and amount of rainfall is becoming increasingly uncertain and the two decades from 1980 have witnessed a trend towards reduced rainfall or heavy rainfall and drought occurring in the same season (GoZ-UNDP/GEF 2009).

Chitiyo and Kasele (2004) assert that the most plausible strategy, therefore, is not only crop diversification but also cultivation of alternative low-input crops such as cassava (*Manihot esculenta Crantz*), which can tolerate the stressful conditions such as drought, acidity and low soil fertility (Asher, Edwards & Howeler 1980; Challinor et al. 2007). Cassava is a potentially high yielding root crop of South American origin. It was introduced into Africa by Portuguese traders in the 16th century and into Zimbabwe from Angola, Malawi, Mozambique, Zaire and Zambia (Carter, Fresco & Jones 1992). No specific production skills are required to grow cassava, which tolerates drought, acidity and low soil fertility (Asher et al. 1980; Hahn, Reynolds & Egbunike 1988). It is against this background that this study sought to probe the potential of cassava production as climate change adaptation strategy in Chilonga in Chiredzi, Zimbabwe.

## Climate change and agriculture

The IPCC (2007) defines climate change as a change in the state of the climate that can be identified (e.g. by using statistical tests) by changes in the mean and the variability of its properties, and that persists for an extended period, typically decades or longer. Adaptation means anticipating the adverse effects of climate change and taking appropriate action to prevent or minimise the damage they can cause or taking advantage of opportunities which may arise (IPCC 2007).

Cassava (*M. esculenta*) is extensively cultivated as an annual crop in tropical and subtropical regions of Africa, Asia and Latin America between 30°N and 30°S (Duangpatra 1988; El-Sharkawy 2006). The total global cassava production in 2009 was about 241 million tonnes, with Africa being the leading producer (FAOSTAT 2010). Cassava is considered a staple root crop for more than 800 million people living in developing tropical countries (Burns, Johnston & Schmitz 2003). About 70% of world cassava root production is used for human consumption either directly after cooking or in processed forms. The remaining 30% is used for animal feed and other industrial products, such as starch, glucose and alcohol (El-Sharkawy 2004). In Brazil and China, cassava is being increasingly used for bioethanol production, while in Nigeria it is a major cash crop earning the country about three billion United States Dollars annually through export of the crop and related products (FAO 2007).

Although cassava requires optimal conditions to achieve high growth rates, it performs well in drought-prone areas and on poor soils and is thus considered one of the most productive tropical crops on marginal lands (Zhang et al. 2008). In seasonally dry and semi-arid environments with less than 700 mm of annual rain, improved cultivars of cassava can give dry root yields of over 3 tonnes/ha (El-Sharkawy 2006). Therefore, cassava is often considered an insurance and hence major food security crop for resource-poor smallholder farmers in marginal lands (Kamukondiwa 1996).

## Conceptual framework

Farmers make decisions at the extensive margin (what to produce at site) and intensive margin (how to produce at a site) in order to maximise economic returns. New perspectives in research on climate change adaptation posit that socio-cognitive factors may be important in motivating individuals to take adaptive actions (Frank et al. 2010). Mitchell (1982) defines motivation as the degree to which an individual wants and chooses to engage in certain specified behaviour. Motivation theory explains the cognitive and psychological processes that drive actions in order to predict behaviour (Mitchell 1982). Maslow’s hierarchy of needs posits that the underlying needs for all human motivation are on five general levels from the lowest to the highest: physiological needs, safety needs, belongingness needs, esteem needs and self-actualisation.

Motivation cannot be observed or measured directly, but can manifest itself through attitudinal and behavioural measures (Ambrose & Kulik 1999). Behavioural manifestations may include active pursuit and use of information and implementation of adaptations. However, identifying both the generic- and climate-specific elements of smallholder farmers’ adaptation behaviour is vital in order to facilitate a societal response to the changes that climate scientists have predicted (Sieber et al. 2010). Tailoring adaptation practices to specific societies may make it possible to offset the adverse impacts of climate change (Fussel 2007). Moreover, assessments of economic adaptations show that in some cases returns on financial investments in adaptation are likely to exceed the returns from a baseline situation (Fussel 2007).

Nevertheless, the availability of information alone remains unlikely to motivate adaptation (Cash et al. 2002; Patt & Schroter 2008). Individuals seek or receive, manage and interpret information in different ways and then use or reject it. Frank et al. (2010) argue that smallholder farmers’ knowledge is largely a synthesis derived from personal experience, local sources of knowledge and external sources of techno-scientific information. How farmers perceive scientists and their knowledge is likely to affect farmers’ use of scientific information in making decisions. Cash et al. (2002) argue that at the core of any decision process involving the creation of knowledge, individuals assess the salience, credibility and legitimacy of available information. Cash et al. (2003) propose that effective management of these three components of information is central to successful knowledge production and the ability to mobilise knowledge for desired actions.

Individuals are not only motivated by information about risk but also by their direct experience with loss and harm brought about by living with hazards (Kasperson et al. 1988). The process of adaptation is affected by perception of risk and evaluation of information, and also by perception of one’s own capacity to adapt or self-efficacy. Motivation theory posits that much of human action can be explained through the concept of perceived self-efficacy, defined by Bandura (1977), as concerned with judgments of how well one can execute courses of actions required to deal with prospective situations. Perceived self-efficacy is positively associated with any behaviour from which a desired outcome is anticipated (Bandura 1977). Frank et al. (2010) argue that smallholder farmers are highly perceptive of climate and its impact on their land and crops. When confronting environmental change, however, local knowledge is not always sufficient in building adaptive capacity. To adapt to change, new information is often needed, in this case information about the changing climate and feasible adaptations of farming practices.

## Cassava production in Africa

Cassava (*M. esculenta*) is the crop with the highest total production in Africa, with 118 million tonnes of productions across the continent in 2010, contributing significant energy input to the population with an average 196 kcal/capita/day in 2008 (FAO 2010; Smit and Skinner 2002). Cassava is a major staple for more than 500 million people in Africa, and is renowned for its drought tolerance and hardiness in stressful environments (El-Sharkawy 2004). From the few studies which have quantified the impacts or responses of cassava to climate change, Jarvis et al. (2012) found out that cassava is the least affected crop when compared with other major staples such as maize, sorghum and millets. Liu et al. (2008) used the Geographical Information Systems (GIS)-based Environmental Policy Integrated Climate (GEPIC) model to evaluate impacts on cassava production across sub-Saharan Africa, finding a change in production to 2030 of -2% to +1% depending on the Special Report on Emission Scenarios (SRES). The results were in agreement with those by Lobell et al. (2008) who found cassava to moderately benefit from climate change by 2030 with an average increase of 1.1% in production from 2000 through the use of statistical models. Jarvis et al. (2012) carried out a research on the impacts of climate change on cassava and compared it with other important African staples such as maize, sorghum, millets, potato, common bean and banana and found that cassava is not affected by soil type and changes in weather patterns. Jarvis et al. (2012) also found that very few studies have focused on cassava when predicting impacts of climate change on crop production, partly because process-based crop models are not accurate or not available at all (Boote et al. 2010; Challinor & Wheeler 2008; Fermont et al. 2009), and partly because most research on climate change impact assessment has focused on better documented staples such as maize, wheat and rice (Bakker et al. 2008).

Case studies in Nigeria and Ghana revealed that in Ghana cassava is a major crop with a hectarage under cassava was 387 000 ha in 1986 and increased to 590 000 ha in 1996 (USAID 2005). During the same periods, cassava production also increased from about 2.9 million tonnes to 7.11 million tonnes. Cassava is by far the largest agricultural commodity produced in Ghana and represents 22% of Agriculture Gross Domestic Product (AGDP) compared with 5% for maize and 2% for rice (Al-Hassan 1989; Day et al. 1996). The 1987/1988 Ghana Living Standards Surveys (GLSS) showed that 1.73 million sampled households (83%) were engaged in cassava production compared with 1.74 million (86%) in maize production (FAO 2005). In Nigeria, Adeniji et al. (1996) postulated that almost all farmers in the main cassava belts of the south-eastern, south-western and central zones grow cassava. Cassava intercropped as a main or minor crop. Cassava produced in Nigeria is used for human consumption and less than 5% is used in industries (Adeniji et al. 1996). Cassava as a food crop fits well into the farming systems of the smallholder farmers in Nigeria because it is available all year round, thus providing household food security (Alderman & Higgens 1992; FAO 2005).

### Cassava production in Zimbabwe

Cassava production in Zimbabwe only takes place on a very limited scale in Mashonaland West and the crop was identified as having potential for increasing diversification in Zimbabwean agriculture (Kleih 1995). Cassava can be used as a food security and industrial crop because it is drought tolerant. There is a need to market cassava as an alternative food to the general populace of Zimbabwe, especially those who live in marginal areas which mainly receive less than 250 mm of rain and temperatures above 23 °C. In Zimbabwe, cassava production is negligible and has not been the focus of agriculture policy because more emphasis is put on production of cash crops such as maize and tobacco, of which these do not do well in areas like Chiredzi.

### Zimbabwe’s policy and regulatory framework for dealing with climate change in the agricultural and food security sector

Zimbabwe under the agriculture and food security cluster is currently developing a climate change policy which emanated from the national climate change response strategy. The policy is meant to increase sustainable intensification and commercialisation of agriculture across agro-ecologies by:

- increasing capacity to generate new forms of practical knowledge, technologies and agricultural support services that meet emerging challenges arising from climate variability and change by supporting research on how indigenous knowledge can be integrated into evidence-based planning premised on good science
- strengthening early warning systems on cropping season quality, rangelands conditions, droughts, floods, disease or pest outbreaks and wildlife movement in order to augment farmer preparedness
- developing frameworks for supporting agricultural specialisation according to agro-ecological zones as well as mechanisms for product exchange, trade and marketing
- increasing the capacity of farmers, extension agencies and private agro-service dealers to take advantage of present and budding indigenous and scientific knowledge on stress-tolerant crop types and varieties
- developing frameworks for promoting science-based crop production and post-harvest technologies and management practices meant to capacitate, identify and promote the embracing of indigenous and improved livestock breeds that agriculture could sustain (Ministry of Environment, Water and Climate 2015).

## Materials and methods

**FIGURE 1:**
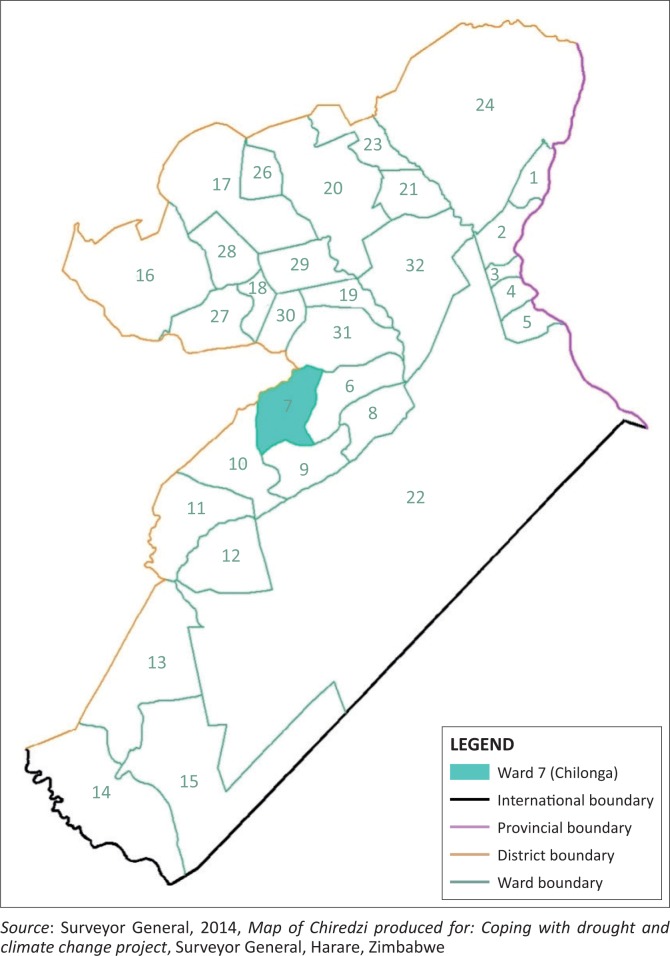
Map of Chiredzi showing location of Chilonga Ward (7).

The research employed a case study design qualitative research methodology which was based on the participants’ own categories of meaning and is responsive to local situations, conditions and stakeholders’ needs. A total of 120 people who were participants of the GOZ/UNDP/EMA Coping with Drought and Climate Change Project in Chiredzi district, Chilonga Ward as shown on Figure 1 during the period 2007–2011 were used as research participants. Agriculture adaptation strategies of the primary participants were included in the study. Purposive sampling technique was used to select key informants and focus groups from Agricultural Technical Extension Services (AGRITEX), Chiredzi Rural District Council (CRDC), Environmental Management Agency (EMA) and the Coping with Drought Project Team that implemented the project. A sample of 40 respondents through stratified random sampling had questionnaires administered to them. Twenty households were used for focus group discussions and these were grouped on gender basis. Purposive sampling was used in selecting key informants. Data were grouped into themes for presentation and analysis.

## Geographical characteristics of Chiredzi District

Chiredzi District is in Masvingo Province, Zimbabwe. It is a vast, semi-arid and drought-prone area downstream of the Save and Runde Catchments with altitude generally below 500 m above mean sea level (Unganai 2011). Chiredzi District borders Mozambique to the east and South Africa to the south – extending over an area of 17 629 square kilometres. Chiredzi has a population of 307 436, and an estimate of 276 842 people live in the rural areas of the district (ZIMSTATS 2012). Average household size is about five people per household. The key features of district include the Save Conservancy, the Gonarezhou National Park and the Manjinji Pan. There are four major rivers: the Save, Runde, Chiredzi and Mwenezi. Chiredzi lies largely in Natural Region V, a region that experiences less than 400 mm rainfall per year in most years (Vincent & Thomas 1960). Natural Region V is an extensive farming region characterised by cattle or game ranching. Lovell (2000) states that temperatures are always very high in summer (± 39 °C), causing evaporation losses of 10 mm – 13 mm per day. Rainfall in Chiredzi decreases from 700 mm in the north to less than 500 mm in the south along the Limpopo Valley (Fewsnet 2007; GoZ-UNDP/GEF 2012).

Annual grasses with scattered shrubs and stunted trees are the main vegetation types (Unganai 2011). Soils are heavy clays. Livestock rearing, of cattle and goats, forms an important component of livelihoods (Wolmer, Sithole & Mukamuri 2002). Sorghum, pearl millet, cowpeas and maize are the dominant food crops grown in the region and red sorghum, cotton and groundnuts are the main cash crops (GOZ/UNDP/EMA 2012).

## Results and discussion

### Demographic data

Table 1 shows that the majority of the respondents were females from the ages 31 to above 51 years old. This has a bearing on their knowledge, experience and enthusiasm in farming.

**TABLE 1:** Gender and age of respondents.

Age group	Male	Female	Total
18–30	3	4	7
31–40	3	10	13
41–50	6	5	11
51+	3	6	9

**Total**	**15**	**25**	**40**

### Marital status of respondents

**FIGURE 2:**
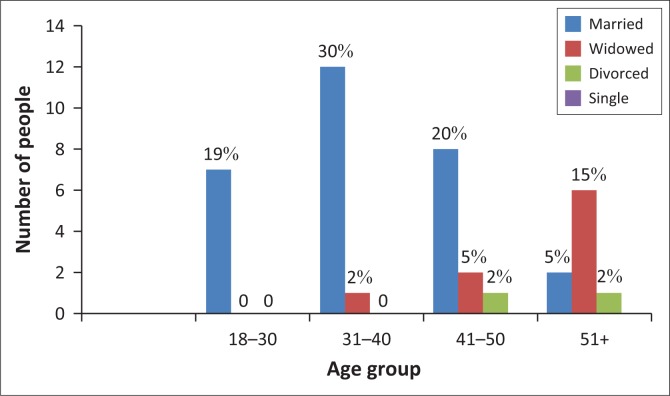
Marital statuses of respondents.

Figure 2 shows that 74% of the respondents are married, 22% are widowed with the age group above 51 having the highest percentage and 4% are divorced. The male-headed households are depicted by the respondents who are married. Because of the patriarchal setup in Chilonga, this has a bearing on the decision-making processes, for example, issues of land tenure or types of crops cultivated.

### Educational qualifications of respondents

**FIGURE 3:**
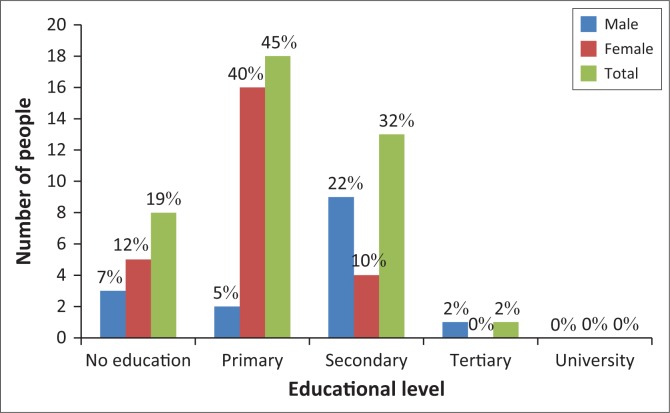
Educational qualifications.

Figure 3 shows that 45% respondents in Chilonga Ward attained primary school education, while most of the females above 51 years old had the highest proportion with no formal education, which is 12%. Figure 2 also shows that 32% of the respondents attained ordinary level. Two percent of the smallholder farmers attained tertiary education. These data show that the literacy levels of all households are very high, and farmers are able to comprehend and to a greater extent appreciate and use information passed on to them by agricultural extension officers.

Table 2 shows that 88% of respondents have resided in Chilonga for over 25 years; this implies knowledge of the climatic conditions. Of the respondents, 93% indicated that they were not willing to relocate; they were born in Chilonga and learned to cope with climatic changes. A total of 7% indicated that they would want to migrate to urban areas or diaspora so as to give back remittances.

**TABLE 2:** Period of residence in Chilonga Ward.

Period of residence in the village	18–30	31–40	41–50	51+	Total	Percentage
0–15	1	-	-	-	1	2
15–20	-	3	1	-	4	10
25+	6	10	10	9	35	88

**Total**	**7**	**13**	**11**	**9**	**40**	**100**

**FIGURE 4:**
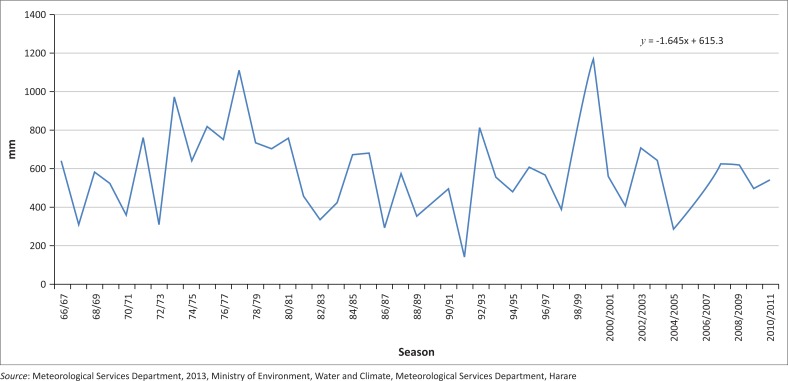
Chiredzi rainfall time series.

The rainfall time series for Chiredzi is shown in Figure 4. The Chiredzi time series for the seasons starting from 1966/1967 to 2010/2011 concurs with the information that was provided by respondents for the period 1980–2014. The season 1999/2000 as depicted in the graph shows that the district received far above normal rainfall.

**FIGURE 5:**
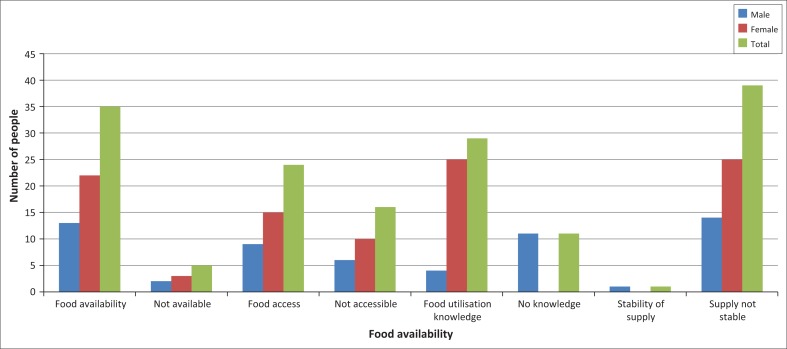
Food availability, access, utilisation and stability of supply.

Figure 5 shows results of food availability, access, utilisation and sustainability of supply differed among respondents. A total of 88% respondents indicated that food was available up to the month of August and 12% indicated food was not available. Sixty percent agreed that food was accessible and 40% indicated that it was not accessible. Figure 5 also shows that 98% of the respondents indicated that they were not food secure. This implies that the smallholder farmers in Chilonga were food insecure. In terms of utilisation, all females and 27% of males indicated that they had knowledge of food utilisation and the other 73% had no knowledge. Twenty-seven percent of male respondents said they also get the information from the clinic when they accompany their pregnant wives to the clinic. DFID (2000) asserts that food security is about the availability of food, access, utilisation (which is the quality and nutrition) and sustainable supply of the food.

### Household socio-economic or livelihood status

**FIGURE 6:**
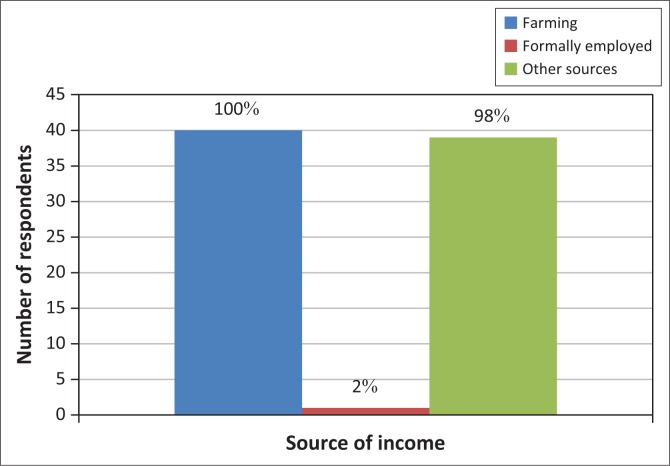
Sources of household income.

The farmers’ responses about their sources of income or livelihood are shown in Figure 6.

Livelihoods are predominantly dependent on rain-fed agriculture and 98% of the respondents rely on farming and income sources such as remittances from relatives in the urban areas and diaspora, market gardening and small-income generating activities as shown in Figure 6. Two percent rely on formal employment and farming. This implies that 98% of the smallholder farmers are vulnerable to climatic extremes. This was confirmed by the District Agricultural Extension Officer that about 98% of the farmers experience food shortages during the period September to March when they will have exhausted the previous season’s harvest.

### Types of crops grown

Figure 6 shows the types of crops grown in Chilonga Ward.

**FIGURE 7:**
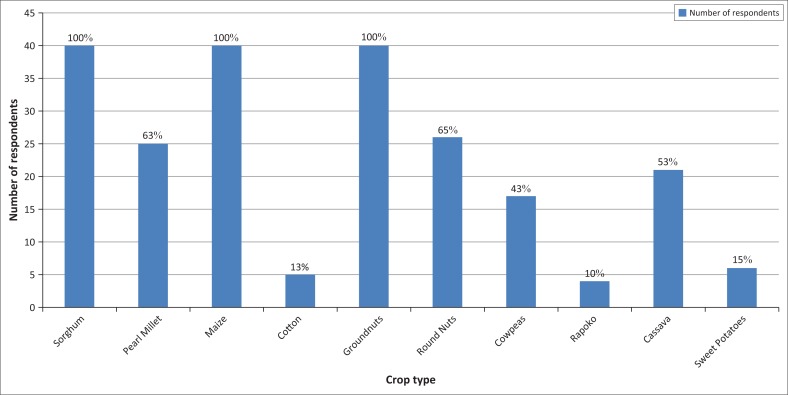
Crops that are grown in Chilonga Ward.

Figure 7 shows that all respondents (100%) are into maize and sorghum cultivation. Groundnuts and roundnuts are cultivated by 63% of the respondents and 53% of the farmers are into cassava production. In Zimbabwe, agricultural policy encourages production of cash crops such as tobacco and maize at the expense of food crops; therefore, the District Council was promoting and encouraging the production of cassava and small-grain crops, especially pearl millet which is tolerant to heat waves and droughts.

### Climate change adaptive strategies employed by the smallholder farmers

**FIGURE 8:**
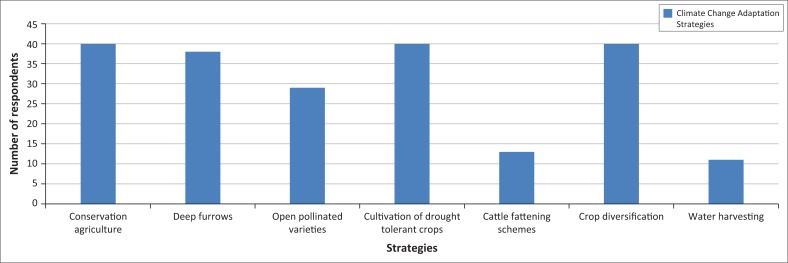
Adaptation strategies in Chilonga Ward.

All respondents practice crop diversification, as shown in Figure 8 as well as conservation agriculture practises such as mulching and filtration pits. They are also cultivating drought-tolerant crops such as sorghum, pearl millet, cassava, rapoko and maize varieties that are drought tolerant. Seventy-three percent said that they also practice cultivation of open pollinated varieties, 33% of the respondents mainly men are practicing cattle fattening with the assistance of Food and Agriculture Organization. Twenty-eight percent of the respondents said that they were practicing water harvesting through digging dead end contours. This implies that farmers in Chilonga are aware of erratic rainfall and high temperatures and therefore engage in the production of crops that are drought tolerant.

### Cultivation of cassava

**FIGURE 9:**
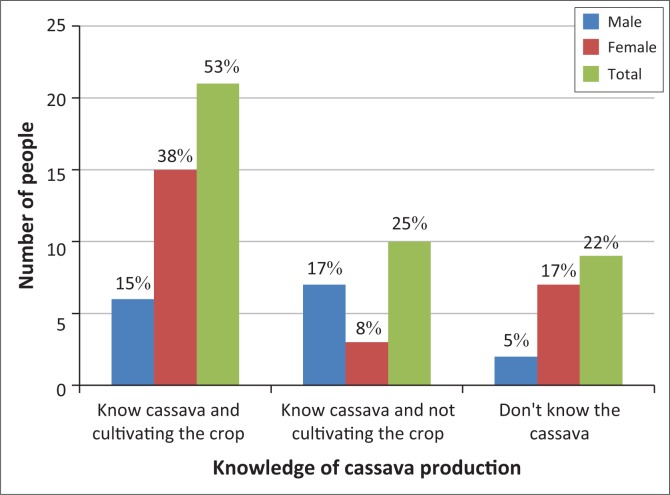
Cassava production in Chilonga Ward.

Figure 9 shows that cultivation of Cassava in Chilonga Ward is still very low, represented by 38% of females and 15% male. Twenty-five percent know cassava and are not cultivating the crop and 22% do not know cassava. Production is still at subsistence level and is for domestic consumption as responded by the 53% that are practicing cassava production. Seventy-eight percent respondents indicated that women were more interested in cassava production than men. This is an indication that women were more motivated to produce cassava and 38% were willing to produce cassava crop on large scale. FAO (2005) asserts that in a study that was carried out in Nigeria, it was found out that women play a central role in cassava production, processing and marketing, contributing about 58% of the total agricultural labour in the South West, 67% in the south-east and 58% in the central zones. FAO (2005) also added that women are said to be entirely responsible for processing cassava which provides them with additional income-earning opportunities and enhances their ability to contribute to household food security. Cassava was introduced as an adaptation option in Chilonga Ward in 2007 by the Ministry of Environment, Water and Climate through EMA and financially supported by United Nations Development Programme (UNDP) through Coping with Drought and Climate Change Programme. By 2010, uptake of cassava remained at less than 1% and the low uptake was because of limited planting material, limited knowledge of the crop and extension services.

### Benefits of cassava

**FIGURE 10:**
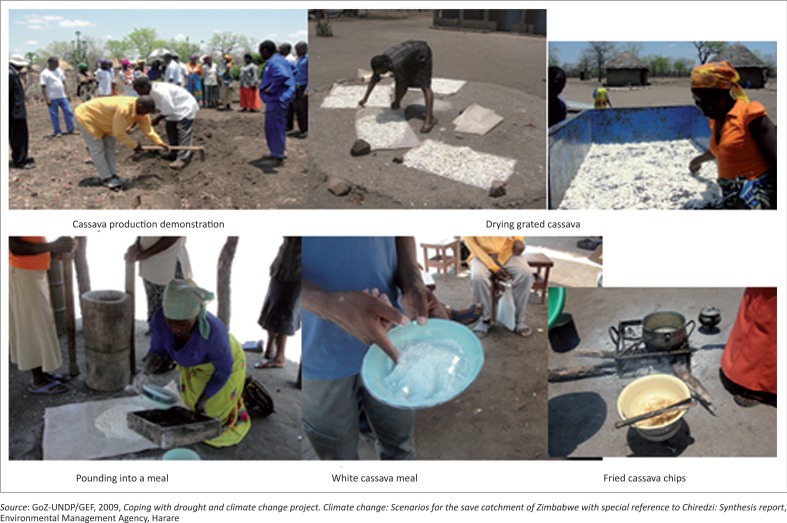
Cultivation and processing of cassava.

Fifty-three percent respondents who are into cassava production said that benefits of cassava include consumption of both tubers and leaves. Tubers can be stored in the ground after harvesting for as long as 4 months. Cassava can be prepared and processed for consumption in many different ways, either by just boiling the tuber or by drying and grounding it into powder which can be used to cook sadza (thick porridge) or make bread and which can also be fried as chips (Ceballos 2011; Gleadow et al. 2009). Figure 10 depicts pictures of demonstration of cultivating cassava, drying of grated cassava, pounding of cassava into meal and fried cassava chips. Thirty-eight percent female respondents were much interested in cassava production as a result of these benefits. This implies that more women will be encouraged to produce cassava.

### Benefits of cassava production

Of the respondents, 78% said that cassava production is not affected by soil type, heat waves, excessive heat or changes in weather patterns. They also agreed that the crop requires enough water when planting and afterwards can survive with little water.

### Limitations of cassava production

Limitations of cassava production that came out from 78% of respondents include little or no knowledge of the crop. Seventy-eight percent of the respondents also indicated that they have limited seed, which is also a limiting factor in large-scale production. Seed is made available through Chiredzi Research Station at a cost and respondents argued that they lacked financial resources to purchase the seed. However, 38% indicated that they were going to get the seed cuttings from the cassava crop in Tamuwanyika Garden in Chilonga. Lack of resources was another limitation that was raised by 78% of the respondents. Fencing material protects the crop from livestock. The respondents echoed that after harvesting of the other crops, cassava will be the only crop that will be green in the fields and, therefore, animals will be attracted to the crop.

## Ethical considerations

Ethical issues were taken into consideration before, during and after data collection process and the subsequent writing process.

## Conclusion

Smallholder farmers remain vulnerable to climate variability and change in Zimbabwe, given their dependence on rain-fed agriculture. The farmers are aware of the changing weather patterns, major causes of change, as well as their impacts within their localities and are thus attributing this to climate change. The perception of smallholder farmers that climate is changing is supported by observables such as changes in rainfall amounts, rainfall distribution, onset and cessation of rains and average temperature. Conservation agriculture and production of drought-tolerant crops that thrive in all soil types are some of the major adaptation strategies by smallholder farmers (Herrera Campo, Hyman & Bellotti 2011). Food security is increased in such areas once drought-tolerant crops such as cassava are grown.

In light of the above conclusions, the study recommends the following:

- There is a need for policy makers to review Zimbabwe’s agricultural policy so that it can encourage production of crops such as cassava. Given the increased frequency of extreme weather events induced by climate change, creating a framework for targeted adaptation is crucial to protect people’s livelihoods in marginal areas such as the Chiredzi District.
- Farmers willing to grow cassava should be assisted with fencing material and seed; this can be on a loan basis with attractive repayment plan such as payment after sale of harvested produce.
- Farmers, EMA, AGRITEX, Meteorological Services Department (MSD), Livestock Extension Officers and the Rural District Council Officers should invest in climate change adaptation strategies, training and initiation of cassava production.
- The issue of cultivating drought-tolerant crops is not yet a policy, but since independence in 1980 the Government of Zimbabwe has been encouraging farmers to grow cash crops such as tobacco. Instead, the drafting of a climate change policy should be expedited so that the growing of drought-tolerant crops such as cassava could become a major thrust. Adaptation is influenced to a certain extent by governmental actions.
